# Supplementary material for: Transient role of the middle ear as a lower jaw support across mammals
Source: eLife. 2020 Jun 30;9:e57860. doi: 10.7554/eLife.57860 (PMC7363448; doi:10.7554/eLife.57860)
Supplement: Figure 6—source data 1. [file elife-57860-fig6-data1.docx]

|  | **Strong Alcian Region**  **Cell Count** | **Weak Alcian Region**  **Cell Count** |
| --- | --- | --- |
| **80µm sections counted** | 8 | 10 |
| **Cell Counts:** |  |  |
| **Minimum** | 28 | 21 |
| **25% Percentile** | 33.5 | 27.75 |
| **Median** | 36 | 28 |
| **75% Percentile** | 40.25 | 36.32 |
| **Maximum** | 44 | 38 |
|  |  |  |
| **Mean** | 36.38 | 29.2 |
| **Std. Deviation** | 4.897 | 6.033 |
| **Std. Error of Mean** | 1.731 | 1.908 |
|  |  |  |
| **Unpaired t test, 2 tail** |  |  |
| **P value** | 0.0152 |  |
| **t, df** | t=2.718, df=16 |  |
|  |  |  |
| **Difference between means** | -7.175 ± 2.640 |  |
| **95% confidence interval** | -12.77 to -1.57 |  |

Supplementary Table 1: Descriptive statistics for mesenchyme cell counts in day. 5 opossum
